# The Management of Health Hazards Related to Municipal Solid Waste on Fire in Europe: An Environmental Justice Issue?

**DOI:** 10.3390/ijerph17186617

**Published:** 2020-09-11

**Authors:** Walter Mazzucco, Claudio Costantino, Vincenzo Restivo, Davide Alba, Claudia Marotta, Elisa Tavormina, Achille Cernigliaro, Maurizio Macaluso, Rosanna Cusimano, Rosario Grammauta, Fabio Tramuto, Salvatore Scondotto, Francesco Vitale

**Affiliations:** 1 Health Promotion Sciences, Maternal and Infant Care, Internal Medicine and Medical Specialties (PROMISE) Department, University of Palermo, 90133 Palermo, Italy; walter.mazzucco@unipa.it (W.M.); claudio.costantino01@unipa.it (C.C.); vincenzo.restivo@unipa.it (V.R.); marotta.claudia@gmail.com (C.M.); fabio.tramuto@unipa.it (F.T.); francesco.vitale@unipa.it (F.V.); 2 Clinical Epidemiology and Cancer Registry Unit, Palermo University Hospital “P. Giaccone”, 90127 Palermo, Italy; 3 College of Medicine, University of Cincinnati, Cincinnati, OH 45267, USA; Maurizio.Macaluso@cchmc.org; 4 Department of Health Services and Epidemiological Observatory, Regional Health Authority, Sicilian Region, 90145 Palermo, Italy; elisaeleonora.tavormina@regione.sicilia.it (E.T.); achille.cernigliaro@regione.sicilia.it (A.C.); salvatore.scondotto@regione.sicilia.it (S.S.); 5 Palermo Health Agency, 90146 Palermo, Italy; rosanna.cusimano.55@gmail.com; 6 Institute for the Study of Anthropogenic Impacts and Sustainability in the Marine Environment (IAS), National Research Council, 91021 Torretta Granitola (Trapani), Italy; grammautarosario@libero.it

**Keywords:** municipal solid waste, environment pollution, health effects, public health, environmental justice

## Abstract

Landfilling should be the last option in an integrated Municipal Solid Waste (MSW) management approach. In the European Union (EU), the policy framework to protect the environment and the public health against the impact of health hazards related to urban solid waste management has been consistently implemented in recent decades. A growing interest in the negative impact of fires in waste landfills on the environment and people’s health was reported in some European countries. In Italy, an increasing occurrence of arsons in MSW and landfills has been reported in recent years. During the summer of 2012, a multi-site arson occurred in the Palermo Municipal solid waste landfill of Bellolampo (western Sicily), giving rise to an environmental emergency of public health concern. Local health authorities reacted by creating an inter-institutional multidisciplinary task force with the aim to implement measures to prevent and control the risk of exposure by delimiting a protection area to be taken under strict monitoring. Environmental and epidemiological investigations were put in place by air, soil, and farm product sampling. A syndromic surveillance of the exposed population was conducted as well. The air monitoring stations system in place detected an increase in the concentrations of dioxins and dioxin-like substances with the PM10 highest emission pick documented within the first 24 h and estimated at about 60 μg/m^3^. Levels of heavy metals above the limits permitted by law were detected in the top- and sub-soil samples collected within the two landfill sampling sites and also in other nearby sites. Non-conforming concentration values of dioxins and dioxin-like substances were detected in samples taken from farms, milk, and water. The health syndromic surveillance did not document any daily increase in the notification of emergency admissions related to acute respiratory diseases or any other health effect potentially related to the waste arson, but these findings were limited by the non-systematic collection of data. The experience reported in the present case report, as declined within the European Union policy framework and in the view of environmental justice, documented the need to structure a permanent collaboration between the different institutional actors involved in environmental and public health protection activities in order to develop specific protocols to manage events related to the occurrence of waste-related environmental emergencies or disasters.

## 1. Introduction

### 1.1. Policy and Regulatory Framework on Waste-Related Environmental Pollution in the European Union

Increased risk to communities from adverse health effects associated with environmental pollution related to the misuse and poor management of municipal solid waste or incinerators has been documented by literature during recent decades [1,2,3,4,5,6].

Waste management represents one of the main challenges for environmental policy making at the international level, and in particular in the European Union (EU), where the policy framework to protect the environment and the public health against the impact of health hazards related to urban solid waste management has been consistently implemented in recent decades [7,8,9]. More recently, waste management policies focused on integrated Municipal Solid Waste (MSW) management and on strategies following a hierarchical approach: waste reduction, recycling or reuse, incineration with energy recovery, and landfilling [10,11]. In this context, landfilling must be considered the last option because it requires space, runs a high risk of leakage into the air, water or soil, and makes less use of the energy content of waste compared to incineration [11,12]. Therefore, EU member states are called to implement waste prevention as well as reusability and recyclability of products in order to reduce the landfilling of the total amount of urban solid waste produced by 10% [13]. Nevertheless, landfilling still represents one of the current waste management approaches within many European countries [14].

Although policies concerning the routine of waste management are in place, to the best of our knowledge, there is a lack in the regulatory and management framework of what is “not ordinary” within the EU countries, including the occurrence of waste-related environmental emergencies or disasters. Nevertheless, a growing interest in the negative impact of fires in waste landfills on the environment and people’s health was reported in some European countries [15,16,17].

Environmental justice aims to guarantee the same degree of protection from health hazards in the environment for everyone, as well as equal access to the decision-making processes that contribute to a healthy living environment [18]. More recently, it has been used synonymously with environmental inequalities; assuming that the distribution of pollution, and the resultant impact on human health and well-being, are generally spread nonuniformly among the population [19].

### 1.2. Institutional Framework Responsible for Environment Protection and Public Health Measures against Environment Pollution in Italy

In Italy, different institutional actors are involved in the protection of both the environment and the general health of the population against the effects of environmental pollutants following a dichotomic approach. Since 2001, according to the devolution principles stated by the reform of the Constitution [20], competences on environment protection and healthcare devolved to the regional level, under the supervision of the central State (Ministry of Health and Ministry of Environment, respectively). To clarify, the State defines the basic levels of health care and services (Livelli essenziali di assistenza - LEA) and the total amount of financial resources necessary for their financing in the view of the National Health System (NHS), while 21 administrative regions and autonomous provinces are responsible for organizing the respective regional health services (RHSs) and guaranteeing the provision of the related services in compliance with the LEAs. Furthermore, the Ministry of Health adopted a “one health” model, consisting of a unitary vision of health that passes through that of animals and reaches man. To this end, it is supported by a network of Experimental Zooprophylactic Institutes (IZSs) that are responsible for epidemiological surveillance and research on environmental and food safety, animal health prevention and promotion, as well as public health issues related to food of animal origin, and of laboratory and diagnostic services in support of food hygiene and food quality control. Moreover, the health care branches of the regional governments provide the organization of prevention and healthcare services on a provincial population-based level and the LEAs delivering through the local health agencies (LHAs).

Similar to the health sector, the responsibility for environmental protection and pollution monitoring is assigned to 21 public territorial environmental protection agencies (EPAs), regional (R-EPA) and provincial (P-EPA), which are part of a nationally integrated network, the National System for Environmental Protection (NSEP), coordinated by the Environmental Protection and Research Institute (ISPRA), a public and independent agency supporting the activities of the Ministry of Environment as defined by the Environmental Codex [21]. The NSEP provides the basic levels of environment protection services (Livelli essenziali delle prestazioni tecniche ambientali—LEPTA) through the regional and provincial EPAs, under the supervision of the Ministry of Environment, so combining the direct knowledge of the territory with the application of the national policies for environmental protection [22,23].

Furthermore, at the local level regional health authorities (RHAs) identify methods and levels of integration between health and environmental policies, by defining programs and operational agreements between LHAs and EPAs related to the protection of the environment, the epidemiological surveillance, and the communication of health-related risks.

Nevertheless, with specific regard to the solid waste management and environment pollution monitoring, the scenario can be different among the Italian administrative regions, whereas the related activities are usually provided by local public or private companies.

### 1.3. The Case of Bellolampo Landfill on Fire

Over the years, Italy has implemented its policy and regulatory framework on waste management according to EU directives with particular regard to an integrated management approach for the disposal of municipal waste [24,25,26]. However, as reported by the Institute for Environmental Protection and Research for 2017, the amount of landfilled waste in Italy was totaled to around 23%, while landfilling still remains the main method of municipal waste disposal in the region of Sicily with percentages of 73% [14]. Of interest, the increasing occurrence of arsons in solid waste treatment plants documented in Italy in recent years [27] highlighted the need to prevent this phenomenon and to investigate, at the same time, the potential health effects related to exposure of pollutants emitted by solid waste management plants on fire laying in proximity to populated areas [28].

Sicily, the first Italian administrative region by area, provides disposal in four landfills of an average 6000 tons of waste daily, produced by the resident population. With 663,401 resident inhabitants, Palermo City is the administrative capital of the region [29]. The Palermo Municipal Solid Waste Landfill (MSW-L) is located on the hidden side of Bellolampo mountain, rising on the south-western border of Palermo City, about 10 km from the city center and 450 m above sea level [30]. The MSW-L collects the urban waste coming from both the metropolitan area and from other small towns situated in the extra-urban area within the borders of Palermo Province, providing for a population of about 1,250,000 resident inhabitants in need [30].

**Figure 1:**
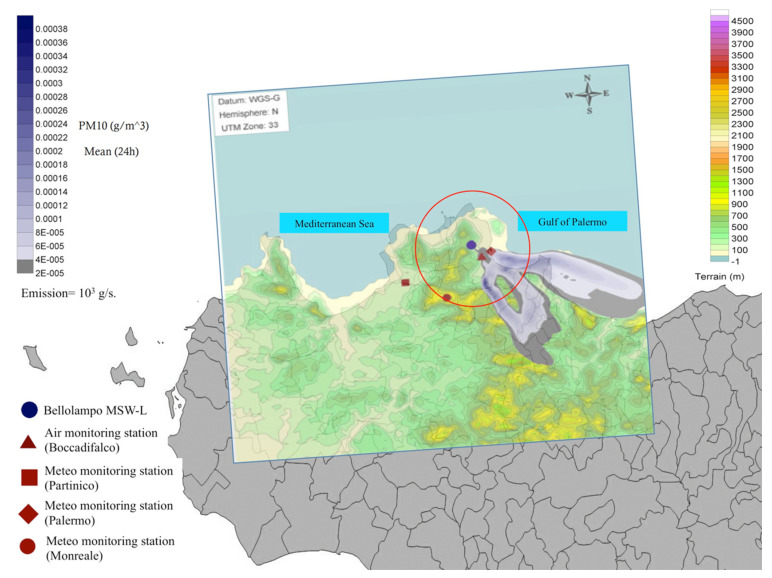
Reconstruction of the Bellolampo landfill environmental scenario. The red circle identifies the “protection area”.

From 29 July 2012 until 16 August 2012 a multi-site arson occurred in the conferring solid waste landfill of Bellolampo [31], involving the fifth most populated area in Italy and giving rise to an environmental emergency of public health concern. The uncontrolled waste combustion, involving a surface of about 120,000 m^2^, emitted consistent particles fallout on the urban area and on the extra-urban rurally populated area surrounding the city (Figure 1). The fire damaged the biogas plant and the leachate treatment system [32] so seriously that on 30 July 2012, the waste transfer to the site was interrupted and the MSW-L was closed.

Against the backdrop of the latest definitions given for environmental justice and according to the current policy framework, we report the results of the management of Bellolampo landfill arson, whereas an integrated public health approach was implemented to protect human health and to counteract the effects of emissions deriving from the municipal solid waste plant on fire.

## 2. Material and Methods

The high health risk perceived by the resident population induced the RHA to create an inter-institutional multidisciplinary task force, integrated with experts in environmental, human, and veterinary public health, with the aim to manage the emergency. On the basis of a preliminary analysis of the scenario, the task force defined an action plan to protect public health and the environment from the emissions generated by the arson. In particular, any action related to environmental and epidemiological investigations, the syndromic surveillance of the exposed population, communication to the general population and any measure to prevent and control the risk of exposure, were put in place.

Following the output of an experimental simulation system, provided by EPA to model the dispersion of air pollutants emitted by the arson (particulate matter and dioxins), using weather parameters, wind direction and geographical characteristics [33,34,35,36] and according to the task force recommendations, the RHA issued a decree declaring the area within the perimeter defined in a circle by a 20 km radius distance from the landfill on fire as a “protection area” at high risk of exposure to dioxins and dioxin-like emissions (Figure 1).

Actions implemented within the “protection area” were: (a) to monitor the concentrations of air pollutants and the dioxins fallout; (b) to put in place a syndromic surveillance of acute diseases or symptoms potentially related to the air pollutants emitted by the arson [37]; (c) to perform soil sampling and to collect organic and inorganic samples to be tested for pollutants contamination, with particular regard for the rural areas.

With the same act, in consideration of the “precautionary principle” [38], the RHA adopted the introduction of public health control measures banning sale and consumption of milk and milk products, meat and eggs, produced from 29 July 2012 and deriving from farms laying in the extended protection area. Mushroom harvesting, grazing and use of forage was prohibited as well. Thorough washing of fruit and vegetables was also recommended to the general population.

Last but not least, the population was strongly recommended to limit outdoor exposure, in particular for frailty subjects as children and elder people.

During the emergency period, press was involved in the work in progress activities of the task force, particularly supporting the health authorities in the communication of the correct messages to the general public.

### 2.1. Monitoring the Concentrations of Air Pollutants and the Dioxins Fallout

Available data on concentrations of air pollutants, routinely performed by the municipal monitoring system [39] consisting of eight stations dislocated across the main axis of the metropolitan area, were extracted with regard to period related to event in study and then transmitted to EPA for further evaluations. In particular, in order to estimate the environmental impact generated by the event EPA was commissioned to monitor the dispersion of contaminants through validated models performing particulate matter <10 microns (PM10) fallout analysis [37]. PM10 concentrations were monitored according to the hypothesis that dioxins and dioxins-like substances can be conveyed by the air particulate [40]. To this end, according to the experimental model of air pollutants’ dispersion [33], the event was divided into three different subperiods: (I) from 29 July 2012 at 2 p.m. to 30 July 2012 at 2 p.m.; (II) from 30 July 2012 at 2 p.m. to 5 August 2012 at 2 p.m.; (III) from 5 August 2012 at 2 p.m. to 16 August 2012 at 2 p.m.

The control air monitoring station (Figure 1) was used to detect variations in the PM10 concentration, being less biased by the traffic emissions than the remaining urban air monitoring stations.

### 2.2. Syndromic Surveillance

According to previous evidence, documenting that exposure to ambient levels of air pollution can be a determinant of an increasing in accesses to emergency department (ED) for acute respiratory and cardiovascular diseases or symptoms [41], the LHAs were commissioned to monitor the number of admissions to EDs potentially related to the air pollutants emitted by the arson. The EDs of the hospitals serving the populations residing in the extended “protection area” were asked to monitor daily the number of cases of asthma, bronchitis, emphysema, or other respiratory symptoms, chest pain, cardiovascular diseases. To this end, EDs were asked to fill a standardized form reporting the number of cases per day, including clinical information related to the selected causes, and the number of total admissions. Every LHA was then responsible for comparing the reported frequency data on ED admissions with the ones observed during the same period in the previous year, and to communicate any excess than the expected number of admissions to the RHA. Furthermore, a correlation analysis between the concentration levels of air pollutants and the number of ED admissions during the arson was planned.

### 2.3. Samples Analysis

Sampling sites were chosen on the basis of the explored dispersion model and also by taking into account the distribution of the resident population in different areas of the city and of the surrounding municipalities. Soil samples were collected at two sites inside the landfill area and in other 100 sites outside the landfill every 2 km up to 20 km radius away from the landfill (sites were identified by letters and progressive numbers). Sampling was conducted at two different levels of depth: topsoil (within 5 cm) and subsoil (at 40 cm or more) [37].

Sample lab analysis were performed by an academic specialized lab center [42] to detect the presence of heavy metals, dioxins as polychlorinated dibenzo*-p-*dioxins (PCDDs) or 2,3,7,8-tetrachlorodibenzo-p-dioxin (TCDD), dioxins-like substances as polychlorinated/polybrominated biphenyls (PCBs/PBBs) or dichlorodibenzo furans (PCDF), polycyclic aromatic hydrocarbons (PAH), volatile organic compounds (VOC) and radio-nuclides.

An IZS with special expertise was enrolled to perform sampling in 34 farms located in the extra-urban area at distances of 1, 3, 5 and 10 km, and within 20 km radius from the landfill. Samples from fodder, milk, and milk products, derived from bovines, sheep, donkeys, and goats bred in farms or home-bred, were tested for pollutants concentration (dioxins, dioxins-like substances, PAH and heavy metals). Water samples from 11 wells laying within the protection area were collected and tested, also.

Sample analysis were performed according to the parameters defined by the Italian legislation and the determination of the recalled pollutants was carried out following the EPA and ISO (UNI) methods [37].

## 3. Results

We report the results of the different investigations suggested by the task force and formally commissioned by the RHA. 

EPA estimated that in the first 24 h (from 29 July 2012, at 2 p.m. to 30 July 2012 at 2 p.m.) about 24 million kg of solid waste and about 54,000 tons in the whole period were on fire [43]. It was also estimated that the amount of PM10 emitted per unit of time ranged between 1.81·10^2^ g/seconds (g/s) and 1.81·10^3^ g/s [33]. The air monitoring stations system detected an increase in the concentrations of dioxins, dioxin-like substances and PCBs [33] with the PM10 highest emission peak documented within the first 24 h (first subperiod) and estimated in about 60 μg/m^3^, with an emission rate of 1000 g/s and a fallout on the ground of 200 μg/m^3^. The average PM10 concentration in the first 24 h was of about 50 μg/m^3^, decreasing to 20 μg/m^3^ from 12 a.m., corresponding to the same average value detected in the 24 h before the arson. During the second subperiod (from 30 July 2012 at 2 p.m. to 5 August 2012 at 2 p.m.) a PM10 average emission rate was of 100 g/s was reported, with a fallout on the ground ranging between 10^−8^ g/m^3^ and 10^−5^ g/m^3^. Lastly, during the third subperiod (from 5 August 2012, at 2 p.m. to 16 August 2012, at 2 p.m.) the PM10 detected an average emission rate of 10 g/s, while the fallout on the ground was always below the legal limits [44]. 

**Figure 2:**
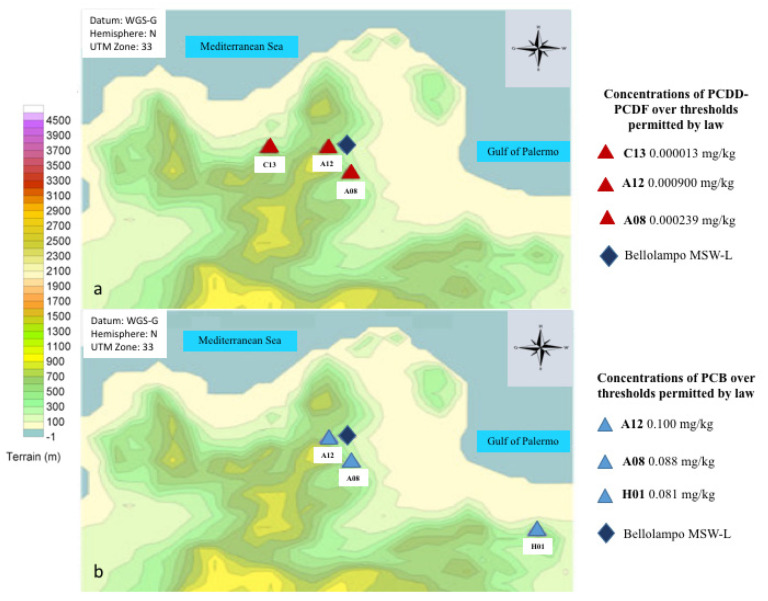
Concentrations of (**a**) PCDD-PCDF and (**b**) PCB over thresholds permitted by law detected in soil samples in the area under surveillance.

Presence of TCCD in soil samples was documented by EPA, but with concentration between 0.1 and 1 ng/Kg (s.s.), resulting under tolerable values. Concentrations of PCDD-PCDF (Figure 2a) and of PCB (Figure 2b) over thresholds permitted by law were detected in three different sites, respectively. Two sites out of three were overlapping for PCDD-PCDF and PCB and were situated in the proximity of the MSW-L (Figure 2). Levels of heavy metals above the limits permitted by law [26] were detected in the top- and sub-soil samples collected within the two landfill sampling sites and also in other nearby sites (data not shown). Overall, findings from sub-soil samples documented a long-term deposition probably independent from the fire disaster.

The analytical report produced by the IZS highlighted the detection of non-conforming concentration values of dioxins and dioxin-like substances [45] in 5 of the 34 monitored farms (locations are not shown because of privacy implications). Moreover, a significant presence of the investigated pollutants was reported in 13 fodder samples, in eight milk samples and in a single water sample. On the contrary, presence of heavy metals resulted negative in all of these collected samples (data not shown).

During the non-systematic health syndromic surveillance conducted by the LHAs, no daily excess in the notification of ED admissions related to acute cases with respiratory diseases or health effects potentially suggestive of exposition to the pollutants emitted by the waste arson, as compared to the ones occurred in the same period of the previous year, was reported to RHA. Therefore, no further analysis correlating concentration levels of air pollutants and the ED hospital admissions during the arson was conducted.

On 7 September 2012, forty days after the arson, the waste plant reopened, while the monitoring plan of the “protection area” was granted an extension through two further decrees [46,47].

On August 2013 one year after the event, the decrees were revoked and every public health measure was ceased [48].

## 4. Discussion

To our knowledge, this is the first case report in Europe dealing with the management of a multi-site arson involving a municipal solid waste landfill with a substantial exposure to a high concentration of air pollutants effecting both the environment and the general population residing in the surrounding areas. Despite the fragmented institutional and regulatory Italian framework, the inter-institutional and multidisciplinary approach resulted in an adequate management of the fire disaster that occurred in the Bellolampo MSW-L during the summer of 2012. However, some criticisms have to be discussed. Since the occurrence of the burning disaster and until one year after, as the emergency was declared over, health authorities had to adopt four different urgent decrees to implement the related measures to manage the event [46,47,48,49], whereas no specific protocol was available against the occurrence of a fire of public health concern within a landfill in proximity of an inhabited area.

Concentrations of air pollutants and admissions to EDs for respiratory issues were monitored over time during the emergency period. This approach was adopted because short-term increasing in concentrations of fine particulate matter in the air was previously documented by literature to be associated with an increasing of ED and hospital admissions for chronic obstructive pulmonary disease, bronchitis, asthma, and chest pain, and of related morbidity and mortality in communities exposed to wildfires disasters [50,51,52,53,54,55,56,57].

Analogous to this, the mentioned evidence should be taken into account also in the case of arson, mainly ascribed to environmental organized crime, or multi-site fires associated with spontaneous combustion related to high temperature peaks, occurring in urban waste management plants.

During the Bellolampo MSW-L arson, despite the documented considerable air pollutants emission and the dioxins fallout, no increase in the notification of admissions to the EDs for respiratory symptoms potentially suggestive of high concentrations exposure was reported as compared to the frequency observed during the same period in the previous year. However, the limits of a non-systematic surveillance should be highlighted. In this context, the importance of the availability of an automated surveillance systems—so that timely information is available during emergencies - has to be stressed [58].

On the contrary, health effects in terms of reproductive, pregnancy and delivery outcomes were retrospectively documented on mothers residing in the proximity of municipal solid waste incinerators [59]: an observational study, conducted to evaluate any potential effect on pregnancies at different gestational ages of pollutants emitted from the Bellolampo landfill on fire by using vital statistics data, highlighted an excess of very preterm and very low birth weight among infants born to mothers exposed to the landfill fire emissions during conception or early pregnancy [28].

Furthermore, even though the air and soil sampling activities documented the presence of dioxins and dioxins-like substances, the interpretation of all the sampling was affected by the lack of previous analysis and results related to the background concentrations values of the pollutants, making any comparisons impossible. Anyway, soil sampling provided evidence of a probable long-term particulate deposition in the area surrounding the Bellolampo MSW-L independently from the arson. Therefore, our results highlighted the importance to perform routine sampling activities in inhabited areas and in breeding and farming sites in proximity to landfills.

The body of the previous evidence underscores how exposure to emissions from solid waste landfill operations may have serious health effects and it supports the need for monitoring potential hazards and health outcomes in the population residing near solid waste management and treatment plants [60].

Of interest is that our experience documented how press and media involvement in the work in progress activities of the task force resulted in effective communication with the exposed population, supporting a high compliance to the public health restriction issued by the health authorities, as reported by previous experiences [61].

The Bellolampo case recalls the perspective of environmental justice [18] with specific regard to the effects, across the Italian country, of a nonuniform spread of environmental pollution among the general population, with higher levels of environmental risk concentrated in disadvantaged population subgroups living in proximity to industrially contaminated sites or to waste management and treatment plants [19,62], and of a nonuniform presence of waste-to-energy (WTE) plants, mainly distributed in the northern area and scarce in the south [12].

More recently, definitions proposed for environmental justice have followed different declinations [19]: exposure and access inequalities, in the view of an unequal distribution of environmental quality between individuals and groups; policy effect inequalities, namely the unequal effect of environmental policies; impact inequalities, i.e., the unequal environmental impact of individuals and social groups with regard to their incomes and/or lifestyles; and policy making inequalities, considered to be the unequal access to environmental policy making. Therefore, the topic of environmental justice could also be applied to the Palermo area, in which several studies showed a high level of impact inequalities [63,64,65].

The declaration from the WHO Regional Office for Europe Ministerial Conference on Environment and Health, held in 2017 in Ostrava, stated the need to prevent and eliminate the adverse environmental and human health effects, costs and inequalities related to waste management and contaminated sites [66]. Nevertheless, no estimates on the overall health impact or assessment on socioeconomic and sociodemographic inequalities in contaminated sites, such as urban waste landfill or waste treatment plants, was available in Europe at that moment [67,68,69]. A survey carried out by the European Environment Information and Observation Network in 2013 reported that waste disposal and treatment are estimated to contribute to more than 37% of contaminated sites [70]. More recently, a study commissioned by the European Industrially Contaminated Sites and Health Network estimated a total of 61,325 disability-adjusted life-years attributable to diseases associated with exposure from landfills [68].

To overcome the scant data availability and to support the decision-making in scenarios as the one reported in the Bellolampo case study, an evidence-based approach, a multi-directional community engagement strategy and consideration of environmental justice concerns have been proposed with regard to the management of hazardous waste sites [70]. Moreover, some mitigating actions have been suggested and, in particular, to promote (a) environmental and epidemiological local monitoring programs on waste management plants to identify inequalities in exposure and disease patterns; (b) the use of the best available technologies to reduce contamination and avoid events of public health concern; (c) the active engagement of citizens in initiatives improving awareness of the health effects of contamination among communities and disadvantaged subgroups [62]. In this framework, the European Union Directives on urban waste management should be applied in order to prevent or mitigate such adverse events as the one documented by the present case report. Many Member States and their regions have not met the target of the *Landfill Directive* [71] obliging them to reduce the amount of biodegradable municipal waste that they landfill to 35% by 2016, as well as to implement the legislative framework for the handling of waste in the community, establishing major principles such as an obligation to handle waste in a way that does not have a negative impact on the environment or human health [72]. In Italy several regions have not met the targets defined by the *Waste Framework Directive* [10], intending to lay down measures to protect environmental and human health by preventing or reducing the adverse impacts of the generation and management of waste and by reducing overall impacts of resource use and improving the efficiency of such use. However, the gap to fill is even greater, if we consider that more recently, the European Commission has adopted the ambitious *Circular Economy Package*, which includes revised legislative proposals on waste to stimulate Europe’s transition toward a circular economy, which will boost global competitiveness, foster sustainable economic growth, and generate new jobs. It consists of an EU Action Plan establishing measures covering the whole cycle, from production and consumption to waste management and the market for secondary raw materials [73]. The revised legislative proposal on waste has set clear targets for reduction of waste and has defined a long-term pathway for waste management and recycling, including (a) the common EU targets for recycling 65% of municipal waste and 75% of packaging waste by 2030; (b) a binding target to reduce landfilling to a maximum of 10% of municipal waste by 2030; (c) the ban on landfilling of separately collected waste; (d) the promotion of economic instruments to discourage landfilling [74].

Therefore, waste management should be improved starting from waste prevention and the reusability and recyclability of products [10]. Urban waste can be transformed into sustainable material, with a vision of protecting, preserving and improving the quality of the environment, protecting human health, ensuring prudent, efficient and rational use of natural resources, promoting the principles of the circular economy, enhancing the use of renewable energy, increasing energy efficiency, reducing the dependence on imported resources, providing new economic opportunities and contributing to long-term competitiveness [12]. In the same direction, the importance of promoting an integrated management of urban solid waste in alternative to landfills, including newly available technologies such as pyrolysis and gasification has to be highlighted [32,75,76].

## 5. Conclusions

The experience reported in the present Italian case report documented the need to structure a permanent collaboration effort between the different institutional actors involved in environmental and public health protection activities, as well as the ones responsible for healthcare, in EU countries. As some relevant criticisms have been documented with regard to the fragmentation of competences between the recalled different institutions, specific protocols should be developed in order to manage and coordinate both routine conditions and extraordinary events related to the occurrence of waste-related environmental emergencies or disasters. Moreover, the continuous monitoring of sensitive sites as such landfills and waste management plants should be promoted and sustained, while penalties against environmental organized crime should be exacerbated at the same time.

In particular, Italy and its regions—especially the ones with warmer climates and at a high risk of arson occurrence—are called to adopt a dedicated health surveillance system and, primarily, to implement the more recent European Union Directives dealing with urban waste management.

In addition, further studies from the perspective of environmental justice should be performed to systematically investigate the effectiveness of environmental and intersectoral actions taken to identify and protect population subgroups documenting a disproportionate environmental burden [62].

## Abbreviations

EU	European Union
MSW	Municipal Solid Waste
NHS	National Health System
RHS	Regional health service
IZS	Experimental Zooprophylactic Institute
LHA	Local health agency
EPA	Environmental protection agency
R-EPA	Regional environmental protection agency
P-EP	Provincial environmental protection agency
NSEP	National System for Environmental Protection
ISPRA	Environmental Protection and Research Institute
LEPTA	Livelli essenziali delle prestazioni tecniche ambientali
RHA	Regional health authority
MSW-L	Municipal Solid Waste Landfill
ED	Emergency department
PCDDs	Polychlorinated dibenzo*-p-*dioxins
TCDD	2,3,7,8-tetrachlorodibenzo-p-dioxin
PCBs	Dioxins-like substances
PBBs	Polybrominated biphenyls
PCDF	Dichlorodibenzo furans
PAH	Polycyclic aromatic hydrocarbons
VOC	Volatile organic compounds
WTE	Waste-to-energy
